# Skeletal Muscle Shape Change in Relation to Varying Force Requirements Across Locomotor Conditions

**DOI:** 10.3389/fphys.2020.00143

**Published:** 2020-03-20

**Authors:** Nicolai Konow, Alexandra Collias, Andrew A. Biewener

**Affiliations:** ^1^Department of Biological Sciences, University of Massachusetts Lowell, Lowell, MA, United States; ^2^Concord Field Station, Department of Organismic and Evolutionary Biology, Harvard University, Bedford, MA, United States

**Keywords:** bulging, locomotion, length change, contraction, compliance, series elastic element

## Abstract

Contractions of skeletal muscles to generate *in vivo* movement involve dynamic changes in contractile and elastic tissue strains that likely interact to influence the force and work of a muscle. However, studies of the *in vivo* dynamics of skeletal muscle and tendon strains remain largely limited to bipedal animals, and rarely cover the broad spectra of movement requirements met by muscles that operate as motors, struts, or brakes across the various gaits that animals commonly use and conditions they encounter. Using high-speed bi-planar fluoromicrometry, we analyze *in vivo* strains within the rat medial gastrocnemius (MG) across a range of gait and slope conditions. These conditions require changes in muscle force ranging from decline walk (low) to incline gallop (high). Measurements are made from implanted (0.5–0.8 mm) tantalum spheres marking MG mid-belly width, mid-belly thickness, as well as strains of distal fascicles, the muscle belly, and the Achilles tendon. During stance, as the muscle contracts, muscle force increases linearly with respect to gait–slope combinations, and both shortening and lengthening fiber strains increase from approximately 5 to 15% resting length. Contractile change in muscle thickness (thickness strain) decreases (*r*^2^ = 0.86; *p* = 0.001); whereas, the change in muscle width (width strain) increases (*r*^2^ = 0.88; *p* = 0.001) and tendon strain increases (*r*^2^ = 0.77; *p* = 0.015). Our results demonstrate force-dependency of contractile and tendinous tissue strains with compensatory changes in shape for a key locomotor muscle in the hind limb of a small quadruped. These dynamic changes are linked to the ability of a muscle to tune its force and work output as requirements change with locomotor speed and environmental conditions.

## Introduction

Skeletal muscle contractions that produce and control *in vivo* movement involve dynamic contractile and elastic tissue strains, as well as changes in muscle shape, that likely interact to influence the force and work production of a muscle. These dynamic changes facilitate the diverse roles of muscles as well as their tendons, which act as biological springs (Roberts and Azizi, 2011). However, studies of the *in vivo* dynamics of skeletal muscle and tendon strains remain largely limited to humans and other bipedal animals, and have rarely examined the broad range of movement requirements met by muscles that may operate as motors, struts, or brakes across the various gaits animals commonly use and conditions they encounter (Dickinson et al., 2000).

When humans and other animals decelerate or move downhill, the function of limb muscles, as a whole, must shift toward eccentric contractions to absorb energy, braking the body’s motion (Gabaldon et al., 2004; Ishikawa and Komi, 2004; Lichtwark and Wilson, 2006; Biewener and Daley, 2007; Konow and Roberts, 2015; Helm et al., 2019). By contrast, when the locomotor task is to accelerate, move uphill, or climb stairs, muscles of the limbs must shift toward concentric contractions to generate greater net positive work (Gillis and Biewener, 2002; Daley and Biewener, 2003; Gabaldon et al., 2004; Hoyt et al., 2005; McGuigan et al., 2009). In comparison, when moving at steady speed on the level, the work requirements of limb muscles are substantially reduced (Cavagna et al., 1964). Indeed, distal limb muscles, for which direct *in vivo* measurements of mechanical work have been obtained, favor more economical force production with limited fascicle strain and work output (Roberts et al., 1997; Biewener and Roberts, 2000). The architecture of these distal muscle–tendon units (MTUs) also favors elastic energy storage and return from their aponeuroses and tendons, with elastic energy savings that substantially reduces muscle work requirements for steady level locomotion (Biewener and Baudinette, 1995; Biewener et al., 1998; McGuigan et al., 2009).

Interactions between a muscle’s fascicles and its aponeuroses and free tendon also significantly affect the force and work dynamics of a muscle and the MTU as a whole. When landing or running downhill, elastic energy stored in the tendon is recycled or absorbed by the stretch of a muscle’s fascicles (Griffiths, 1991; Konow et al., 2012; Konow and Roberts, 2015). The rapid stretch of the MTU initially accommodated by the stretch of the muscle’s tendon provides a “mechanical buffer” that allows the muscle’s fascicles to stretch more slowly, reducing the risk of eccentric muscle injury (Griffiths, 1991; Reeves and Narici, 2003; Roberts and Azizi, 2010; Roberts and Konow, 2013). When accelerating or running uphill, elastic energy stored in tendon and aponeuroses can be released more rapidly than the positive shortening work of the muscle’s fascicles, increasing MTU power output as a whole (Daley and Biewener, 2003; McGuigan et al., 2009). As observed for the human medial gastrocnemius (MG) (Lichtwark and Wilson, 2006), interactions between the muscle’s fascicles and elastic tendon not only allow the muscle’s fascicles to perform shortening work during both incline and decline locomotion but also likely allow the fascicles to operate at shortening velocities that maximize their power output. Similarly, during stair ascent and descent human MG fascicles exhibit differing length change behaviors from the MTU, presumably due to tendon compliance (Spanjaard et al., 2007).

In addition to interactions between fascicle and tendon (or aponeurosis) strains of MTUs, skeletal muscles also undergo changes in width and thickness during contractions. Based on *in situ* muscle studies, 3D changes in muscle shape have been hypothesized to result from the interplay of muscle fiber forces and the resistance of connective tissue to fiber rotation and contractile bulging (Azizi et al., 2008; Holt et al., 2016). Specifically, at low muscle forces, fiber shortening was observed to be associated with the rotation of fascicles and an increase in pennation angle, so that the muscle thickness increased. However, at high muscle forces, fibers shortened with less rotation, and muscle shape change was interpreted as favoring an increase in width versus thickness (Holt et al., 2016). Although these prior studies suggest that 3D changes in muscle shape may significantly affect the force and work output of the MTU as a whole, the phenomenon of 3D bulging remains poorly studied *in vivo*.

Measurements of muscle shape change under dynamic locomotor conditions across a range of gait (speed) and slope conditions are therefore needed to better understand how these variables may affect the force and work output of a muscle. Prior ultrasound studies have shown that the human MG resists bulging in thickness (reduced fascicle rotation) during high-force contractions when cycling at high crank torque (Dick and Wakeling, 2017). We hypothesize that a similar trade-off between width and thickness bulging will occur in the rat MG during unrestrained *in vivo* locomotion; specifically, we predict that the rat MG will undergo widthwise bulging during high-force contractions, minimizing fascicle rotation to augment whole muscle force, along with reductions in widthwise bulging, and increases in thickness, to increase whole muscle shortening velocity during low-force contractions. We test this hypothesis by obtaining fluoromicrometry measurements (Konow et al., 2015; Camp et al., 2016) of muscle shape change, fascicle strain, and tendon strain, as well as measurements of force from the rat MG MTU across a comprehensive range of gait and slope conditions.

## Materials and Methods

### Animals, Training, and Surgery

Five adult male Sprague Dawley rats (275–320 g) obtained from Charles River Laboratories (Wilmington, MA, United States) were housed at the Concord Field Station (Harvard University) and studied in compliance with IACUC- and USDA-approved protocols. Rats were trained 5 days per week to locomote on a DC motorized treadmill (10 cm wide × 60 cm long, equipped with a textured rubber belt to prevent slipping) for 2–3 weeks, until they were able to move steadily at all gaits at each particular speed (walk: 0.25 +0.02 m s^–1^; trot: 0.51 +0.03 m s^–1^; gallop: 0.75 +0.06 m s^–1^) and slopes (downhill: −20°, level, and uphill: +20°). Animals were encouraged to maintain position on the treadmill by gently tapping or briefly gusting their hindquarters with compressed air. The treadmill was customized with a carbon fiber radio-translucent base (Airex C70-40 0.25″ foam core; Dragonplate.com; Elbridge, NY, United States) beneath the rubber treadmill belt to facilitate dorsoventral X-ray imaging (see below). The animals moved within an enclosure constructed of 0.318 mm Plexiglas sidewalls.

**FIGURE 1:**
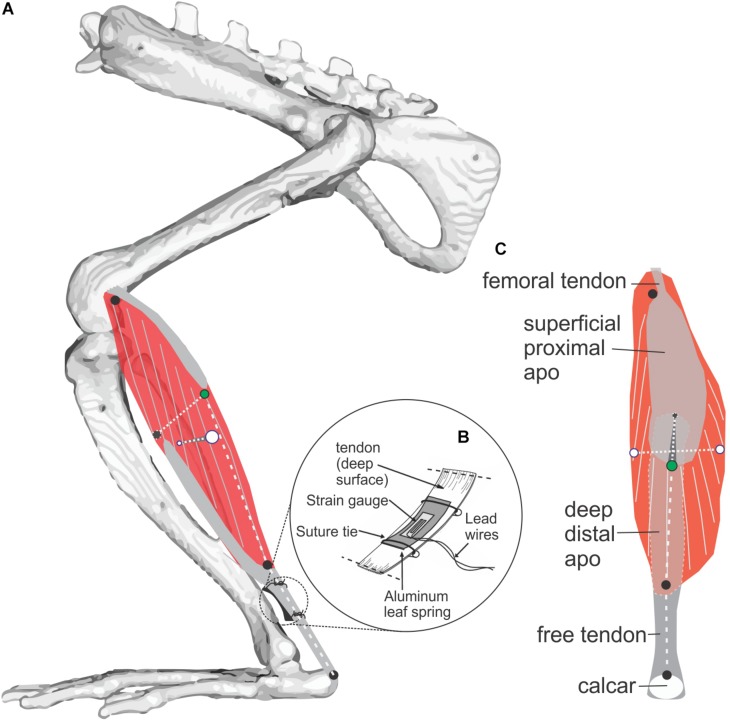
Study muscle and instrumentation. **(A)** Diagram of the rat MG (medial view) showing architecture of muscle fibers (gray on red muscle belly) and aponeuroses (gray). Radio-opaque marker placements at superficial, deep, medial, and lateral muscle surfaces is shown with black and green markers used to measure muscle thickness, and white markers used to measure muscle width. Length of the muscle belly and its tendon were measured using markers along the muscle’s line of action at the positions indicated by three black circles. **(B)** Placement of a foil strain-gauge transducer on the muscle’s tendon (Richards and Biewener, 2007) to measure MG force (separate experiment; *N* = 3, see Figure 3). **(C)** Superficial (posterior) view of the MG showing marker locations.

Following treadmill training, the rats were anesthetized (isoflurane: 2–4% induction and 1–2% maintenance, administered at 0.8–1.0 L O_2_ min^–1^ through a small nose cone) and their left hind limb prepped for surgery (fur removed, followed by betadine scrub). Under sterile surgical conditions, the belly of the left MG was exposed by means of a 2–3 cm medial skin incision and blunt dissection of the superficial-most surrounding fascia. Although dissection of the overlying fascia may influence muscle shape change due to fascial interactions (Huijing et al., 2003; Maas and Sandercock, 2010), this is unavoidable to obtain direct recordings of length change using the methods employed here and in normal physiological conditions, as studied here, these effects are believed to be small (Maas and Sandercock, 2008). Seven sterilized radio-opaque tantalum spheres (0.5–0.8 mm; Abbott Balls, West Hartford, CT, United States) were implanted into the muscle using a trochar constructed from 21- or 18-gauge hypodermic needle stock, respectively, equipped with a stainless steel rod plunger. Markers were placed at the following locations: mid-belly lateral, medial, superficial, and deep epimyseal surfaces; distal and proximal aponeuroses, and distal end of the Achilles tendon, at its calcar attachment (Figure 1; and see Supplementary Figure S1 without overlying labels). The proximal origin of the MG was exposed by reflecting the overlying medial hamstring muscles to implant the proximal aponeurosis marker. The rat MG is a unipennate muscle with fascicles running from the superficial proximal aponeurosis to the deep distal aponeurosis. The superficial muscle belly marker positioned at the distal end of the proximal aponeurosis was aligned with the distal MG fascicles that insert on to the deep aponeurosis at the location of the distal aponeurosis marker. These markers, therefore, provided measurements of distal fascicle strains in relation to changes in muscle width, thickness, whole muscle length, and tendon length. We should note that our measurements assume no change in the alignment of the epimysium at these marker locations, and thus the transverse plane of muscle thickness and width, with respect to the axis of overall muscle tension over a contraction cycle across gait–slope conditions. Additionally, recent bi-planar ultrasound measurements of human tibialis anterior 3D shape change indicate that changes in cross-sectional shape may vary over proximo–distal regions of muscle length during isometric contractions (Raiteri et al., 2016).

After closing the medial skin incision with 4-0 nylon suture (Ethicon, Inc., Somerville, NJ, United States) the rats were allowed 5–7 days to recover with analgesics (Flunixin meglumine, 2 mg/kg) administered every 12 h for the first 2 days to minimize post-operative pain and inflammation.

### Fluoromicrometry Recordings

**FIGURE 2:**
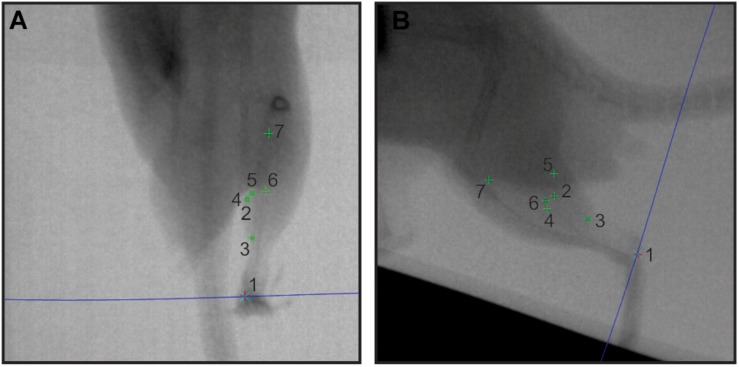
Sample *in vivo* X-ray data. A pair of X-ray stills (**A** – dorsoventral; **B** – lateral) showing digitized radio-opaque markers in the rat MG (numbers 1–7). The blue epi-polar line indicates the direct linear transformation prediction for marker 1, calcaneus. Other markers are: 2, lateral muscle margin; 3, distal myotendinous junction; 4, medial muscle margin; 5, superficial aponeurosis tip; 6, deep aponeurosis tip; and 7, proximal myotendinous junction.

To obtain *in vivo* recordings of muscle–tendon shape and length change, the treadmill was positioned so that the dorsoventral and mediolateral movements of the rat subject (Figure 2) could be captured by two orthogonally arranged X-ray C-arms (OEC 9400, Radiological Imaging Services, Hanover, PA, United States) operating at 70–90 kVp and 2.0–3.8 mA, each equipped with a high-speed video camera (Photron PCI-1024; 1024 × 1024 pixels, with 1/2000 s exposure; San Diego, CA, United States). X-ray settings were established to provide the most ideal contrast of the tantalum beads with respect to surrounding musculoskeletal structures. Walking was captured at 125 frames s^–1^, while trotting and galloping were recorded at 250 frames s^–1^. A minimum of five strides per rat were recorded for each gait–slope combination.

**FIGURE 3:**
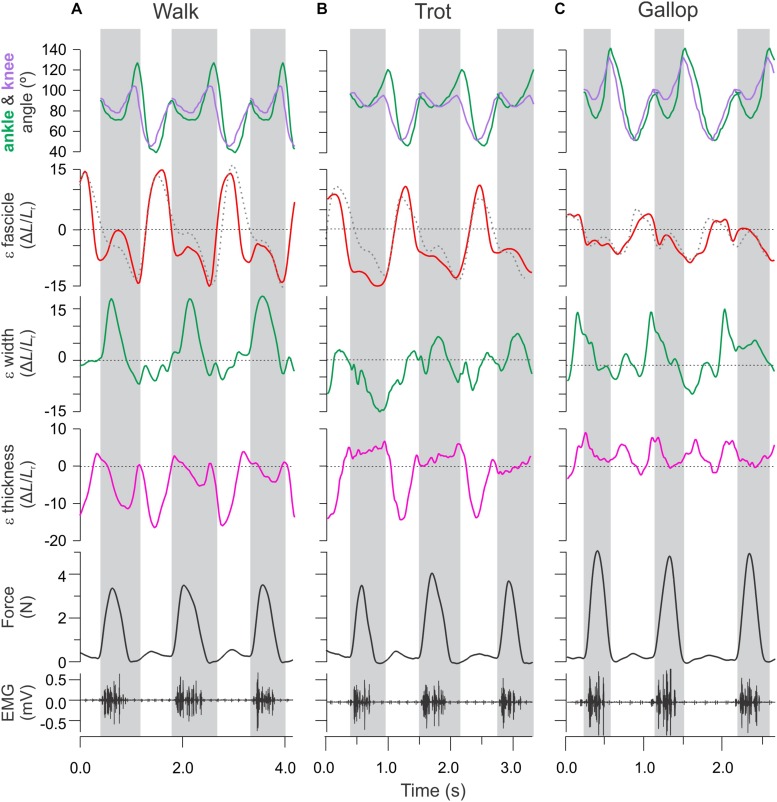
Time varying strain, force, and activation patterns for the rat MG in relation to ankle and knee joint angle changes. Data from **(A)** level walking, **(B)** level trotting, and **(C)** level gallop. Force, activation, and sonomicrometry data are from a different animal than the joint kinematics and strain data. Strain data measured in this study using fluoromicromery (shown as solid traces) are comparable to fascicle length measurements using sonomicromery, the gold-standard approach (shown as dashed traces).

Videos were inspected for quality assurance, including image quality, and presence of all markers in both views. No images were rejected due to quality. Only those that did not provide imaging of the rat MG in both X-ray views over a complete stance phase of a stride cycle were not analyzed. This process reduced our sample to the strides indicated in Table 1. The three-dimensional coordinates of each marker for each trial were reconstructed from digitized XY locations of the marker in each pair of frames using XMAlab software v.5.1 (Knörlein et al., 2016). The relevant distances between marker pairs were then extracted following a recently established fluoromicrometry workflow (Konow et al., 2015; Camp et al., 2016; Figure 2), the accuracy and precision of which has been validated (see Camp et al., 2016). Time-varying changes in distal fascicle length, muscle length, thickness, width, and free tendon length were calculated and exported to IgorPro (Wavemetrics, Inc., Lake Oswego, OR, United States) for subsequent analysis, together with the timing (based on frame number) of the stance (toe–down) and swing (toe–off) phases of each stride (Figure 3).

**TABLE 1:** Strides analyzed per rat for each gait–slope combination and muscle dimension.

		Slope combination								
		
		Down	Down	Down	Level	Level	Level	Up	Up	Up
Rat	Dimension	walk	trot	gallop	walk	trot	gallop	walk	trot	gallop
11	Length	5	7	5	5	5	6	1	5	4
11	Width	5	7	5	5	5	6	4	–	4
11	Thickness	5	7	5	5	5	6	4	–	4
12	Length	2	7	4	6	–	4	5	7	5
12	Width	5	7	4	6	–	4	5	7	5
12	Thickness	5	7	4	6	–	4	5	7	5
16	Length	–	5	4	5	–	2	6	–	5
16	Width	2	5	4	5	–	2	6	–	4
16	Thickness	2	5	4	5	–	2	6	–	5
18	Length	–	–	2	–	–	6	–	–	2
20	Length	2	–	–	6	3	–	3	3	–
20	Width	2	–	–	7	3	–	3	3	–
20	Thickness	2	–	–	7	3	–	3	3	–
21	Length	7	4	3	6	1	4	4	3	3
21	Width	6	4	1	7	1	4	4	3	3
21	Thickness	6	4	1	7	1	4	4	3	3

**FIGURE 4:**
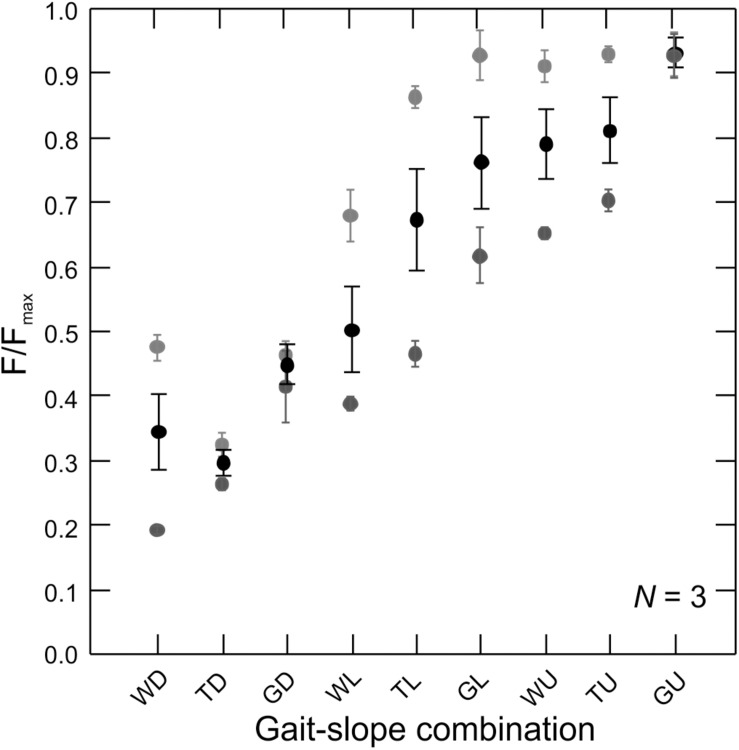
Force production by the rat MG across gait–slope combinations. Gray tones are subjects (*N* = 3). Circles represents the mean of all recorded strides for a given subject (Table 1). Error bars are mean ± SD. There is a statistically significant relationship between muscle force production and gait–slope combination (LMM: *t*_2_,_336_ = 22.58; *p* < 0.0001).

Lengthening and shortening strains of the MG fascicles were measured for the stance phase of each stride, where the muscle is activated (Eng et al., 2019). Fascicle strains were normalized by dividing length changes by the average fascicle length measured for each animal during level walking over a complete stride (stance and swing). We report shortening strains as negative and lengthening strains as positive values (Figure 4). Shortening and lengthening strains measured between successive image–frames were then summed over each stance phase to quantify net fascicle shortening and lengthening. Active lengthening of fascicles was based on the timing of muscle activation (EMG) and onset of force development (Figure 3). Similar measurements of net changes in whole muscle width, thickness and length, and tendon strain were obtained by summing length changes measured between successive image–frames over the stance phase of each stride across all gait–slope combinations.

### Muscle–Tendon Force and EMG Measurements

In a separate set of experiments, *in vivo* electromyography (EMG) and MG–tendon force measurements were obtained from three adult male Sprague-Dawley rats of similar weight (275–315 g) trained to move over the nine gait and slope conditions. MG muscle–tendon recordings were made using a custom-fabricated “leaf–spring” tendon force transducer (∼1.7 mm wide × 6.5 mm long; Figure 1 inset), following the design used to record muscle–tendon forces from plantaris longus in *Xenopus laevis* (Richards and Biewener, 2007). Briefly, the leaf–spring tendon transducers were constructed from two thin aluminum strips obtained from the wall of a beverage can, glued together along their length using cyanoacrylate adhesive (Loctite Corp., Avon, OH, United States), with a small metal foil strain gauge (FLK-1-11, Tokyo Sokki Kenkyujo, Ltd., Tokyo, Japan) bonded using cyanoacrylate adhesive to the concave surface of the curved leaf–spring (Figure 1). Following this, 36-gauge lead wires were soldered to the strain gauge and insulated with epoxy. Two short lengths (∼6 cm) of 4-0 silk suture (Ethicon, Inc., Somerville, NJ, United States) were also epoxied to the surface of either end of the transducer (to anchor the convex surface of the transducer against the tendon when implanted). The entirety of the transducer was then coated with M-Coat A (polyurethane curing agent, Micromeasurements, Inc., Raleigh, NC, United States) to further seal and insulate the circuit, eliminate adverse tissue reaction, and minimize tendon chafing. The shallow curvature of the aluminum functions as a leaf–spring to allow tensile muscle force transmitted via the tendon of the muscle to be measured by the strain gauge as the leaf–spring is deflected under the applied load.

Bi-polar offset hook EMG electrodes (1 mm bared tip; 1.5 mm spacing) were constructed from insulated 0.1 mm silver wire (California Fine Wire, Grover Beach, CA, United States), implanted mid-belly, and sutured to the muscle’s epimyseal surface with 5-0 silk. The EMG electrodes and leaf–spring tendon force transducers/lead wires were disinfected in Cetylcide^TM^ solution (Cetylite, Inc., Pennsauken, NJ, United States) and rinsed repeatedly in sterile water before implantation. Lead wires were provided slack and passed subcutaneously to an epoxy insulated, custom-designed micro-connector (GM-6, Microtech, Inc., Boothwyn, PA, United States) that was anchored to the skin overlying the neck using 3-0 Vicryl suture (Ethicon, Inc., Somerville, NJ, United States).

Muscle shape changes, fascicle strains, and tendon strains were related to changes in force requirements across gait and slope combinations based on *in vivo* recordings of MG–tendon force (Figures 1, 4).

### Implant Verifications and Measurement Calibrations

Following the *in vivo* experiments, subjects were deeply anesthetized (Isoflurane 4% in induction chamber), and euthanized (intra-cardiac overdose injection of sodium pentobarbital). For subjects used for bi-planar high-speed X-ray experiments, the implanted hind limb was dissected free, carefully de-gloved, fixed in 10% phosphate-buffered formalin, and soft-tissues of the whole limb were contrast-stained in a 4% alcoholic solution of Phosphomolybdenum acid, and micro-CT scanned (Bruker 1670). Volume segmentation of scans enabled us to measure the error in distance measurements caused by markers not being lodged precisely at the muscle borders (for muscle width and thickness) or myotendinous junctions (fascicle and whole muscle length). Where needed, segment corrections using the residual distances obtained from the scans were performed on the raw distance data.

For subjects used to obtain muscle–tendon force recordings, a 4-0 silk suture was tied to the calcar, which was then severed from the foot to release the distal MG attachment. The suture was attached to a load-cell (Kistler 9203) and a series of pull calibrations were obtained, ensuring that the voltage levels recorded *in vivo* were reached. By regressing the time-varying load-cell (N) and strain gauge (V) signals against each other, we obtained the slope of the linear relationship with *R*^2^ > 0.96 (Supplementary Figure S2), which was used to calibrate our *in vivo* tendon strain gauge measurements to force (N) (Gabaldon et al., 2004; Richards and Biewener, 2007). The quality of fit to a linear relationship between force and voltage output of the transducers used here matches that obtained from the anuran plantaris tendon (Richards and Biewener, 2007), as well as “E”-shaped buckle transducers used to measure muscle tendon forces in cats (Walmsley et al., 1978), guinea fowl (Daley and Biewener, 2003), goats (Walmsley et al., 1978; McGuigan et al., 2009), wallabies (Biewener and Baudinette, 1995), and strain-force recordings of calcified turkey tendons (Gabaldon et al., 2004).

### Statistics

We used linear mixed models (Systat Software, Inc., San Jose, CA, United States), factoring gait–slope combination (a categorical variable with nine states) and individual as random effects, and intercepts of the model as fixed effect, to determine the effect of gait and slope on our stride–specific, dependent variables; MG peak stance force; fascicle strain, tendon strain, and muscle shape (width and thickness changes) (Table 2). Means ± SD were also calculated for each variable for all gait and slope combinations.

**TABLE 2:** Summary linear mixed model statistics for relationships between gait–slope combinations and the fixed and random effects factored.

	Fixed effect (intercept)			Random effect (G–S combo)			Random effect (individual)		
			
	Estimate (lower–upper 95% CI)	SE	*P*	Estimate (lower–upper 95% CI)	SE	*P*	Estimate (lower–upper 95% CI)	SE	*P*
*F*_MG_	0.341 (0.232–0.45)	0.056	***	0.089 (0.081–0.096)	0.004	***	−0.006 (−0.009 to −0.004)	0.001	***
*V*_muscle_	−0.670 (−1.133 to −0.207)	0.235	**	−0.089 (−0.161 to −0.018)	0.036	**	0.001 (−0.008 to 0.009)	0.004	n.s.
ε_thickness_	−0.689 (−0.731 to −0.647)	0.021	***	−0.013 (−0.017 to −0.009)	0.002	***	−0.030 (−0.037 to −0.023)	0.003	***
εwidth	–0.598 (−0.671 to −0.524)	0.037	***	0.017 (0.010–0.024)	0.003	***	0.014 (0.009–0.018)	0.002	***
ε_tendon_	0.094 (0.050–0.138)	0.014	**	0.006 (0.001–0.008)	0.002	***	−0.033 (−0.004 to −0.001)	0.001	**

Please see section “Results” for *t*-statistics. ***P = 0.001; **P = 0.01.

## Results

Measurements of *in vivo* MG–tendon forces (normalized to the peak force recorded for each animal, *F*_max_) across the nine gait and slope combinations showed a statistically significant pattern (LMM: *t*_2_,_336_ = 22.58; *p* < 0.0001) of force increase from downhill walking to uphill galloping (Figure 4). Moreover, MG–tendon force increased with gait changes from walking, via trot, to gallop (*t*_1_,_452_ = 72.28; *p* < 0.0001) and with changes in slope from downhill, via level, to uphill (*t*_1_,_336_ = 20.21; *p* < 0.0001).

**FIGURE 5:**
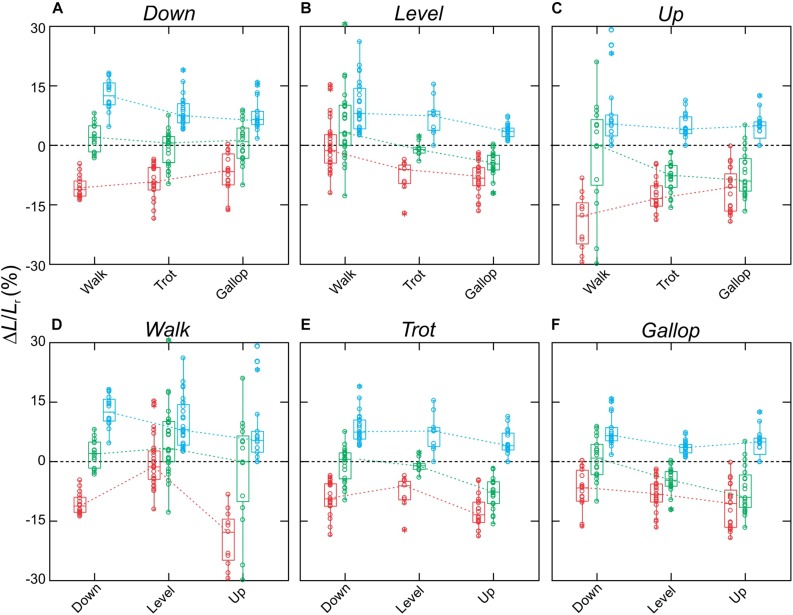
Fascicle strains for the rat MG. Data shown **(A–C)** across slopes, with respect to gaits, and **(D–F)** across gaits, with respect to slopes. Boxes show median with 75th and 25th quartiles, whiskers are the data range for all strides of all subjects pooled. Dot-density circles are stride-specific data points. Red indicates shortening strains, blue are lengthening strains, and green are net strains.

MG fascicle strains measured via fluoromicrometry across slope and gait conditions exhibited generally consistent patterns when compared across slopes with respect to gaits (Figure 5A) but more complex patterns when compared across gaits with respect to slope (Figure 5B). MG fascicles underwent active lengthening strains during early stance where the center-of-mass is being decelerated and the ankle joint flexes and shortening strains during the second half of stance where the center-of-mass is being re-accelerated and the ankle joint re-extends (Figure 3). As a result, net fascicle strains were generally less than either lengthening or shortening strains for all gait–slope combinations, except for level walk (Figure 5). Net MG fascicle strains ranged from 2.0 ± 3.5% (mean ± SD) to 0.8 ± 5.3% for all downhill gaits, from 3.5 ± 11.2% to -4.5 ± 1.9% during level gaits, and from 0.1 ± 7.4% to -8.0 ± 4.2% for uphill gaits. Level and uphill walking gaits exhibited the greatest variability in MG fascicle strains, consistent with the less steady locomotor behavior exhibited by the rats during walking. Except for walking, both trotting and galloping gaits exhibited increased net MG fascicle shortening as animals transitioned from downhill to level to uphill gait (Figure 5B).

**FIGURE 6:**
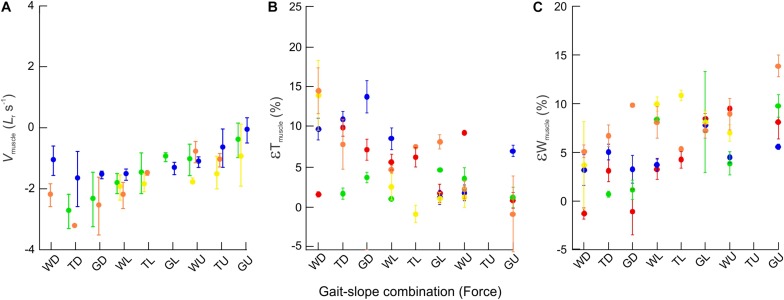
Bulging dynamics of the rat MG. Scatter plots showing changes across gait–slope combinations in **(A)** muscle contraction velocity (normalized to resting muscle fascicle lengths per second) **(B)** εT_muscle_, and **(C)** εW_muscle_. Colors represent subjects and each dot indicates the mean of all strides for a particular gait–slope combination, with error bars giving one standard error around the mean. There are statistically significant relationships between all three response variables with respect to gait–slope combinations (LMM: *V*_muscle_; *t*_1_,_336_ = 22.58; *p* < 0.001. εT; *t*_1_,_140_ = −6.90; *p* < 0.001. εW; *t*_1_,_154_ = 5.08; *p* < 0.001).

When compared across all gait–slope combinations, the pooled data exhibited a significant decrease in changes of rat MG thickness (*t*_1_,_140_ = −6.90; *p* < 0.001), increase in changes in MG width (*t*_1_,_154_ = 5.08; *p* < 0.001), and a reduction in MG muscle belly contraction velocity (*t*_1_,_336_ = 22.58; *p* < 0.001) with increased force, as driven by changes in gait and slope conditions that alter the force generating requirements of the muscle (Figure 6).

**FIGURE 7:**
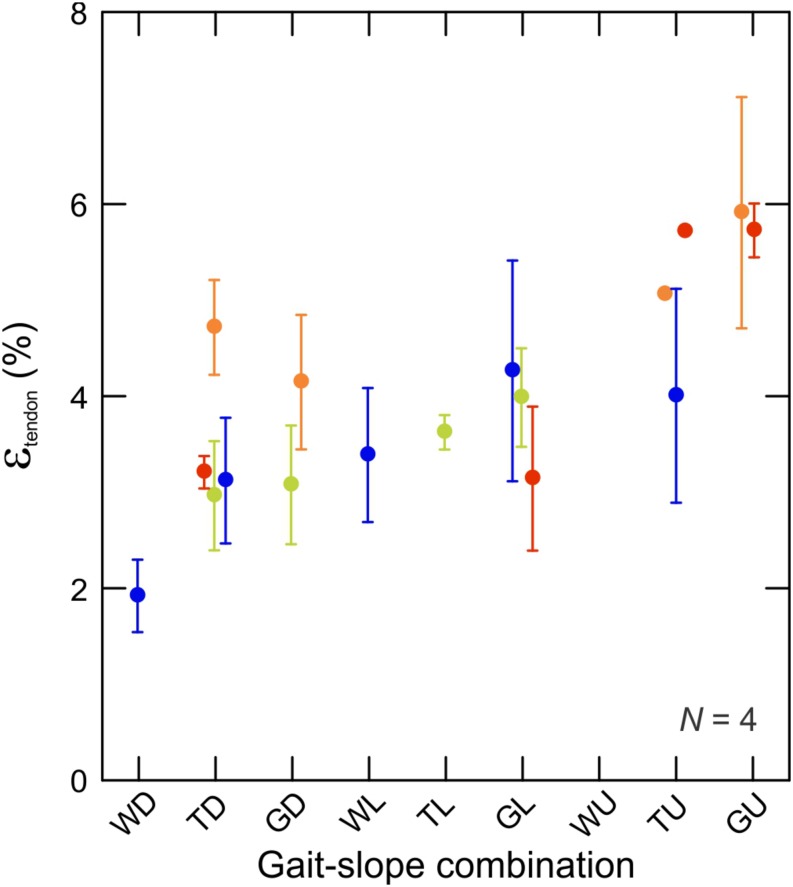
Tendon strain during the force-rise period of stance. Data are mean ± SEM and colors indicate subjects (*N* = 4). There is a statistically significant relationship between tendon strain and force production (by proxy of gait–slope combination) (*t*_2_,_336_ = 22,58; *p* < 0.0001).

Fluoromicrometry measurements of MG tendon strain exhibited a significant increase (*t*_2_,_67_ = 2.53; *p* < 0.014) with respect to gait–slope combination (Figure 7) that paralleled the increase in MG–tendon force (Figure 3). Such an increase is expected, given the passive elastic properties of tendon. MG tendon strains increased from 1.95 ± 0.50% (mean ± SD) during downhill walking to 5.82 ± 1.12% during uphill galloping.

## Discussion

Our fluoromicrometry results for the MG muscle–tendon unit of healthy young adult rats, obtained across a range of gait and slope conditions that require changes in MG–tendon force, support our hypothesis that the rat MG preferentially bulges in width during high-force contractions (uphill gallop) to augment whole muscle force, but preferentially bulges in thickness to increase whole muscle shortening velocity during low-force contractions (downhill walk). Similar shifts in muscle belly shape change were observed within the human MG across a range of low torque to high torque pedaling conditions (Dick and Wakeling, 2017) and are consistent with earlier *in situ* force–velocity measurements of the turkey LG showing increased fascicle rotation to facilitate whole muscle shortening during low-force contractions, but restricted fascicle rotation during high-force contractions (Azizi et al., 2008). In the case of pedaling, shape changes of the human MG were force-dependent but not velocity-dependent across different torque and cadence conditions (Dick and Wakeling, 2017). For the rat MG, we observe force-dependent, as well as whole muscle velocity-dependent, dynamic changes in muscle shape.

Fascicle MG strains recorded via fluoromicrometry were generally similar to the patterns of strain recorded in separate experiments using sonomicrometry across gait and level versus incline conditions (Figure 3; Eng et al., 2019), offering further validation of fluoromicrometry as a viable method for assessing fascicle strain patterns in relation to muscle shape changes (Camp et al., 2016). Although changes in muscle belly thickness and width were correlated with changes in muscle force linked to the varying gait–slope combinations, fascicle strain patterns varied more consistently with changes in slope than with gait. Fascicle lengthening strains during the first half of stance (force development) and shortening strains during the second half of stance (force relaxation) generally exceeded net fascicle strains across nearly all gait–slope conditions (except for level walk). Overall, net fascicle strains (measured distally within the muscle) were small (±1.2%) across gaits during downhill locomotion, varied from net lengthening strain during level walking (3.5 ± 11.2%) to net shortening strain during level galloping −4.5 ± 1.9%, and exhibited the largest net shortening strains during uphill trotting (-8.0 ± 0.4%) and galloping (−9.0 ± 0.6%). The limited net MG fascicle strains during level gait and increase in net shortening strain during incline trot and gallop generally match the patterns observed based on sonomicrometry (Eng et al., 2019). These patterns indicate limited net fascicle strain and work output of the rat MG during level locomotion, but increased shortening and work during uphill gait.

Tendon strains showed a statistically significant relationship with force development as strains increased with the increase in force requirements associated with the different gait–slope conditions. This finding indicates an increase in the role of elastic energy return from downhill walk to uphill gallop and adds to the debate about the role of tendon compliance in small mammal locomotion (Ker et al., 1988; Bullimore and Burn, 2005). The strains we recorded for the rat Achilles tendon *in vivo* match well with the data from recent materials testing of the same tendon (Javidi et al., 2019). Because MG fascicle strains showed little evidence of net lengthening across all gait–slope conditions, energy stored in the MG tendon during force development is presumably recovered to power limb and body movement, rather than being dissipated by doing work on the muscle (via fascicle stretch; i.e., “elastic backfire”; *sensu* (Roberts and Azizi, 2011); in contrast to the buffering of rapid stretch and energy absorption observed for turkey landings from drops of different heights (Konow and Roberts, 2015) and for landings from human jumps (Werkhausen et al., 2017; Werkhausen et al., 2018; Helm et al., 2019; Hollville et al., 2019).

Our fluoromicrometry results for young adult rat MG shape change are also consistent with those reported for healthy young versus old rats based on *in situ* sonomicrometry and ergometry measurements of fascicle and whole muscle length change and muscle force (Holt et al., 2016). Whereas the MG of healthy young rats exhibited variable gearing, increased MG connective tissue stiffness in aged rats resulted in no change in muscle gearing (ratio of whole muscle velocity: fascicle velocity). Although measurements of muscle thickness and width were not obtained directly by Holt et al. (2016), the increased gearing for low-force isotonic contractions was interpreted as being facilitated by an increase in muscle thickness (resulting from fascicle rotation), as we observe here. Under high-force isotonic contractions, the reduction in gearing was interpreted as a preferential change in muscle width to preserve force output of the muscle by restricting a change in muscle thickness (Holt et al., 2016). Again, our results for muscle shape change during unrestrained locomotion across gait–slope combinations requiring changes in muscle force requirements support the *in situ* pattern observed for the MG of young adult rats, in contrast to the pattern observed for aged rats.

Prior ultrasound work on humans has characterized muscle architectural dynamics, initially in 2D (e.g., Aggeloussis et al., 2010; Giannakou et al., 2011) and recently in 3D, using bi-planar ultrasound (Raiteri et al., 2016) or by shifting one ultrasound probe to measure muscle thickness and width alternatively in carefully controlled dynamometer studies (Randhawa and Wakeling, 2018). Measurements of MG thickness bulging are relatively common (Dick and Wakeling, 2017; Hodson-Tole and Lai, 2019), and reported thickness strains of approximately 14% for the human MG are in-line with our measurements from the rat MG. By contrast, transverse (widthwise) muscle bulging is less commonly measured *in vivo* (Raiteri et al., 2016; Randhawa and Wakeling, 2018), and available data appear to vary with muscle architecture and function. Widthwise strains of approximately 28% for the human MG (Randhawa and Wakeling, 2018) are in line with our measurements from the rat MG. However, in a recent study of the human tibialis anterior (a bi-pennate ankle dorsiflexor), there was no relationship between contraction intensity and muscle widthwise bulging, which overall was limited (Raiteri et al., 2016). It is also noteworthy that compared to the rat and human MG, the human TA exhibits very different shape–change dynamics in relation to muscle and fascicle length–change as contraction intensity increases (Raiteri et al., 2016).

### Potential Limitations of the Study

Our study of how *in vivo* shape changes of the rat MG interact with variation in muscle force to adjust muscle work and power output across gait and grade conditions depends on the simplifying assumption that the transverse plane across which width and thickness length changes were measured remained uniform with respect to overall changes in muscle length. Additional markers would need to be implanted to evaluate whether this assumption remains valid during *in vivo* skeletal muscle contractions. Whether or not this is the case may also vary across muscles having differing architecture. Surgical interference of overlying fascia to implant the small tantalum markers may also have compromised the integrity of the intact muscle in its native state, influencing the dynamics of the muscle’s bulging during contraction. However, fluoromicrometry measurements of muscle length (and shape) change based on small trochar-implanted tantalum spheres has considerable advantage compared with more invasive dissections required to access muscles and implant sonomicrometry crystals—another method commonly used to obtain direct *in vivo* recordings of muscle length change—which require penetrating the epimyseal lining of a muscle and anchoring lead wires to the muscle’s surface. Ultrasound-based methods (e.g., Aggeloussis et al., 2010; Giannakou et al., 2011; Raiteri et al., 2016; Dick and Wakeling, 2017), while clearly the best and most suitable method for *in vivo* assessments of human skeletal muscle length and shape change, depend on reliable anchoring of the ultrasound probes to minimize skin movement artifacts relative to the underlying muscle and adequate image quality obtained from superficial muscles. Further, ultrasound methods are not feasible for smaller animal studies, which also allow direct measurements of muscle–tendon force.

Finally, our use of a leaf–spring tendon force transducer depends on a linear and stable calibration of force with respect to voltage output from the transducer’s strain gauge. By calibrating the transducers immediately after obtaining *in vivo* muscle–tendon force recordings, we minimize the risk that our transducer calibration is not an accurate recording of *in vivo* force. The calibrations of this custom-designed transducer (Supplementary Figure S2) match well the linear fits obtained using “E”-shaped buckle transducers (Walmsley et al., 1978; Biewener and Baudinette, 1995; Daley and Biewener, 2003) and strain gauges bonded directly to calcified tendons (Gabaldon et al., 2004), and their design allows for recording *in vivo* forces in very small tendons, which are not amendable to bulkier “E”-shaped buckle transducers.

## Conclusion

Our measurements of muscle shape change dynamics during unrestrained locomotion across differing gait–slope combinations based on 3D X-ray imaging and fluoromicrometry support the findings of past studies (Azizi et al., 2008; Holt et al., 2016) that have inferred muscle shape change based on *in situ* force–velocity measurements and how changes in force output affect fascicle rotation and the gearing of pennate muscles. Thus, increased gearing by bulging of muscle thickness, allowing fibers to rotate under low-force conditions, versus reduced gearing by preferential bulging of muscle width to favor higher force output generally appears to apply to both controlled *in situ* muscle contractile experiments as well as the dynamics of muscle shape change that occur during human pedaling (Dick and Wakeling, 2017), similar to the unrestrained locomotion across gait and slope conditions studied here. Our data on how interactions among muscle shape, fiber length change, muscle force, and connective tissue behavior affect the dynamics of muscle work across varying motor tasks expand our understanding of how the dynamics of these interactions broaden the functional repertoire of whole muscles in relation to their underlying force–velocity and force–length properties. This understanding will benefit musculoskeletal modeling approaches by increasing their abilities to account for how dynamic changes in muscle shape influence whole muscle shortening and work.

## Data Availability Statement

Data used in this study are available from the authors upon request.

## Ethics Statement

The animal study was reviewed and approved by Harvard University’s FAS Animal Care and Use Committee.

## Author Contributions

NK and AB contributed to the study design. NK contributed to the data acquisition. AC contributed to the tracking. NK and AC contributed to the data organizing, analyses, and statistical testing. NK, AC, and AB contributed to the manuscript draft, editing, and approval.

## Conflict of Interest

The authors declare that the research was conducted in the absence of any commercial or financial relationships that could be construed as a potential conflict of interest.

## Supplementary Material

FIGURE S1 Full-frame version of the image pair used for Figure 2. Digitalization markers were removed so that radio-opaque markers can be seen. Each image is 1024 × 1024 pixels.

FIGURE S2 **(A)** Time-varying signals obtained during pull-calibration of strain-gauge (ε[V]) against the Kistler load-cell (N) for one of the rats. **(B)** Three examples of regression lines (thick, black) fitted to the data for force-rise (see panel **A**), showing the equation and *R*^2^ (≥0.96) for each fit.
