# Supplementary figures and images for: Skeletal Muscle Shape Change in Relation to Varying Force Requirements Across Locomotor Conditions
Source: Front Physiol. 2020 Mar 20;11:143. doi: 10.3389/fphys.2020.00143 (PMC7100385; doi:10.3389/fphys.2020.00143)

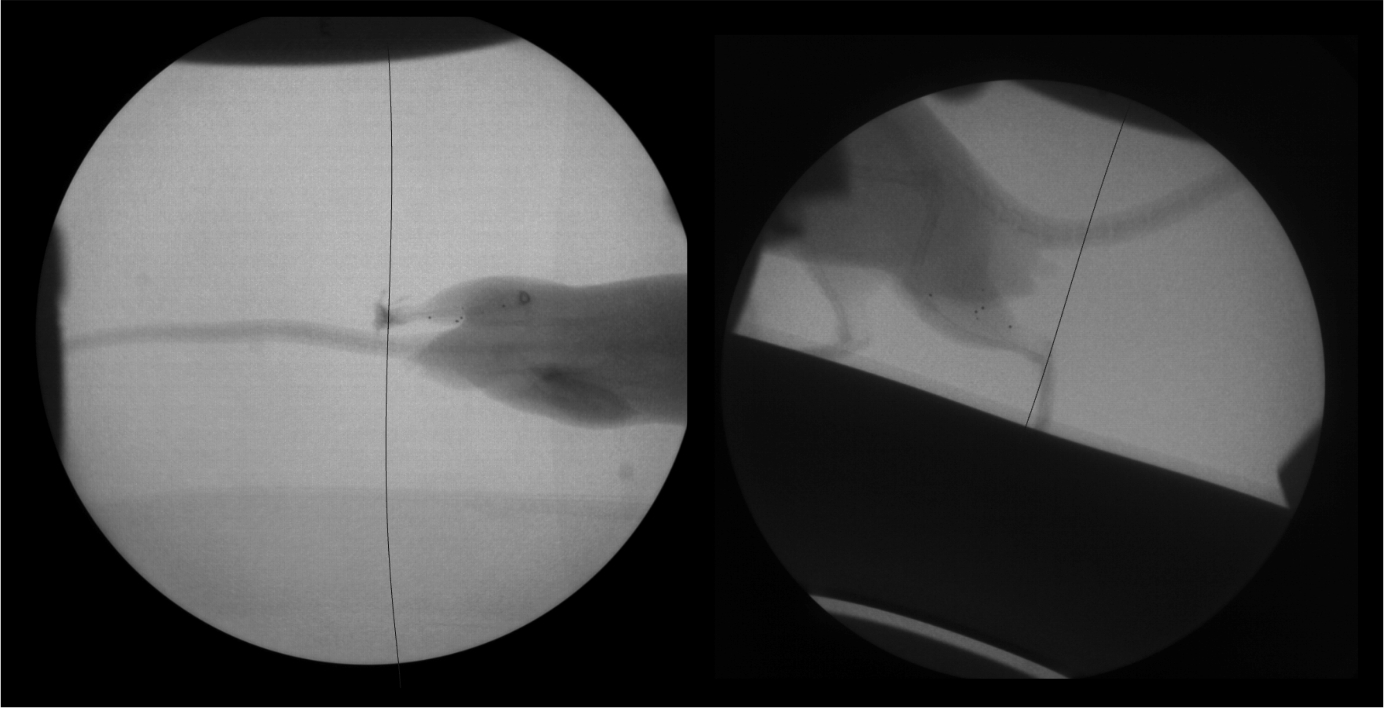

Supplement: FIGURE S1 — Full-frame version of the image pair used for Figure 2. Digitalization markers were removed so that radio-opaque markers can be seen. Each image is 1024 × 1024 pixels. [file Image_1.jpg]

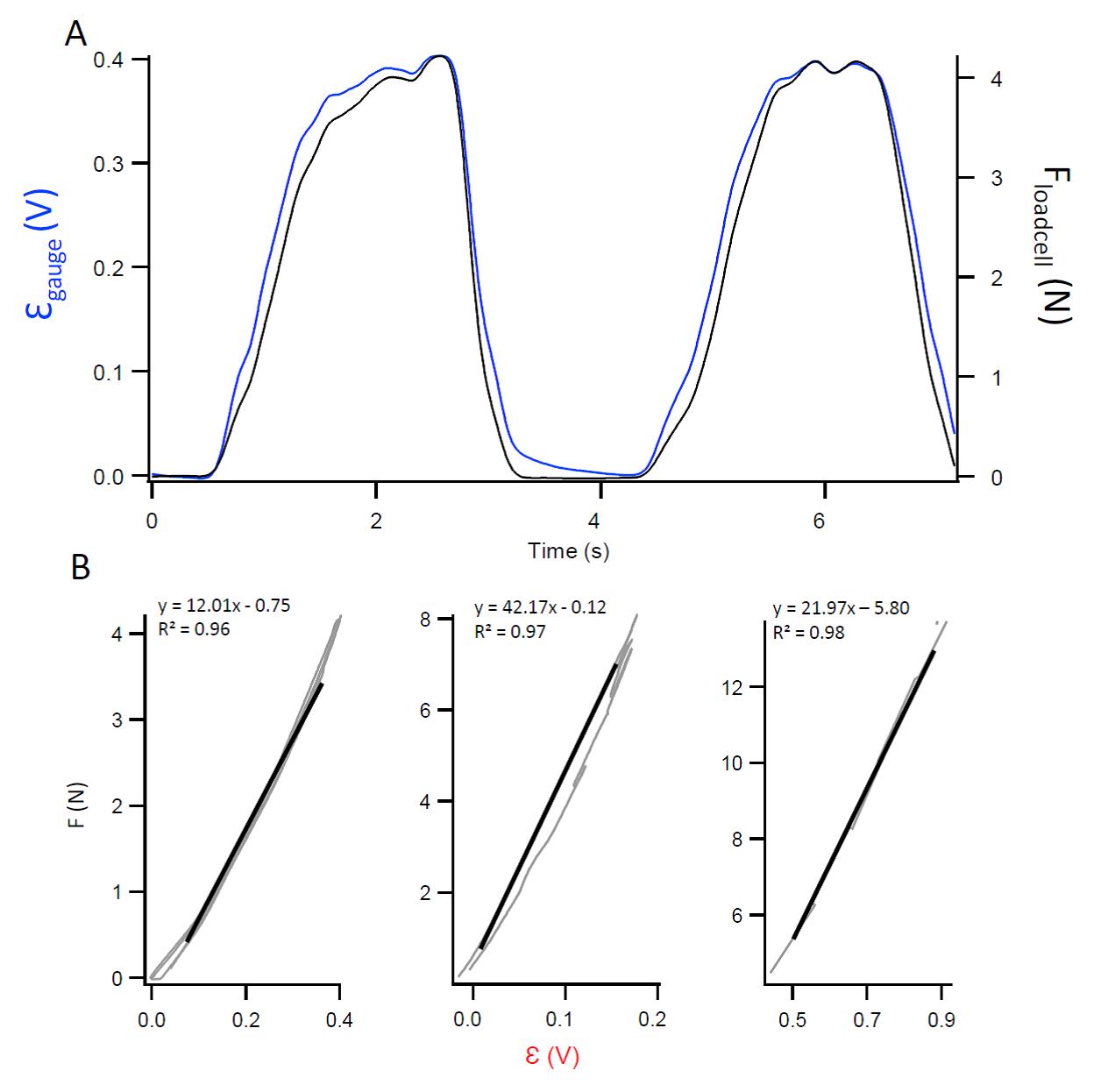

Supplement: FIGURE S2 — (A) Time-varying signals obtained during pull-calibration of strain-gauge (ε[V]) against the Kistler load-cell (N) for one of the rats. (B) Three examples of regression lines (thick, black) fitted to the data for force-rise (see panel A), showing the equation and R2 (≥0.96) for each fit. [file Image_2.jpeg]
